# Pharmaceutical lobbying in Brazil: a missing topic in the public health research agenda

**DOI:** 10.1590/S1518-8787.2016050006508

**Published:** 2016-11-24

**Authors:** Francisco José Roma Paumgartten

**Affiliations:** I Departamento de Ciências Biológicas. Escola Nacional de Saúde Pública. Fundação Oswaldo Cruz. Rio de Janeiro, RJ, Brasil

**Keywords:** Orphan Drug Production, legislation and jurisprudence, Drug Industry, Drug Evaluation, Drug Approval, Products Registration, Health Priorities, Conflict of Interest, Lobbying

## Abstract

In the US, where registration of lobbyists is mandatory, the pharmaceutical industry and private health-care providers spend huge amounts of money seeking to influence health policies and government decisions. In Brazil, where lobbying lacks transparency, there is virtually no data on drug industry expenditure to persuade legislators and government officials of their viewpoints and to influence decision-making according to commercial interests. Since 1990, however, the *Associação da Indústria Farmacêutica de Pesquisa* (Interfarma – Pharmaceutical Research Industry Association), Brazilian counterpart of the Pharmaceutical Research and Manufacturers of America (PhRMA), main lobbying organization of the US pharmaceutical industry, has played a major role in the advocacy of interests of major drug companies. The main goals of Interfarma lobbying activities are: shortening the average time taken by the Brazilian regulatory agency (ANVISA) to approve marketing authorization for a new drug; making the criteria for incorporation of new drugs into SUS (Brazilian Unified Health System) more flexible and speeding up technology incorporation; changing the Country’s ethical clearance system and the ethical requirements for clinical trials to meet the need of the innovative drug industry, and establishing a National Policy for Rare Diseases that allows a prompt incorporation of orphan drugs into SUS. Although lobbying affects community health and well-being, this topic is not in the public health research agenda. The impacts of pharmaceutical lobbying on health policies and health-care costs are of great importance for SUS and deserve to be investigated.

## INTRODUCTION

Lobbying is the act of attempting to persuade legislators and government officials of the validity of a particular viewpoint and, by doing so, seeking to influence decisions according to certain interests or causes. The word lobby finds its roots in the Latin term ‘*laubia*’ or ‘*lobia*’, employed for a covered walk in the Middle-Ages monasteries, where monks used to meet and talk with each other^8^. At the beginning of the 17^th^ century, the term entered into the political vocabulary when it was used to designate the gathering of members of parliament, journalists, and influence-seekers in the hallways (lobbies) of legislative chambers to make conversation^8^. A distinction is most often made between advocacy, a broad term encompassing a range of activities that a person or organization undertakes to influence policies, and lobbying, a type of specific advocacy for influencing legislators and government decision makers^a^.

Lobbying as such is not illegal, nor is it necessarily immoral. As long as it is transparent and does not go beyond persuading by argumentation, lobbying is part of the democratic process. Nonetheless, it becomes unethical when lobbyists, in one way or another, provide benefits to legislators and public officials to misdirect decision-making in Congress and government. Under the influence of unethical lobbying, legislators and government officials are prone to favor special private interests instead of serving public interests. In fact, in many cases, it is hard to distinguish unethical lobbying from a thinly veiled bribery and corruption.

Advocacy towards the promotion of private interests is generally done by organized groups of people who are paid directly or indirectly by industries, corporations, or associations of companies. In the USA, Canada, Australia, Austria, and some other countries, lobbying is regulated and the registration of professional lobbyists is mandatory^b^. In the EU parliament, where a Transparency Register was set up, and in the UK, Germany, and a few other countries, registration of lobbyists is voluntary^b^. In Brazil, where lobbying has not been regulated so far, a bill (PLS 336/2015) setting up a mandatory lobbying register is currently under consideration by the Federal Senate^c^.

Although the lobbying done by health-care providers, health insurance companies, and pharmaceutical and health product industries may influence community health and well-being, public health researches have seldom addressed this type of lobbying and its consequences. A search in Medline database (as for June 5^th^, 2015) revealed 4,690 documents for the broadest searching term “lobbying”, 129 for “lobbying AND US”, and 10 for “lobbying AND Brazil”. For the alternative search strings “big pharma lobbying”, “pharmaceutical lobbying”, “pharmaceutical lobbying and US”, and “pharmaceutical lobbying AND Brazil”, 4, 106, 9, and 1 documents^3^, respectively, were found.

Pharmaceutical and health product industries spent amounts towards a whopping US$3,103,588,993 on lobbying efforts in the US between 1998 and 2015, and the sector occupies the top position in the rank of industrial sectors according to total expenditures in lobbying (Figure)^d^. Not surprisingly, pharmaceutical lobby has successfully accomplished most goals that favored private interests at the expenses of general public interests.^1^ An example along this line is the 2003 Medicare Prescription Drug Improvement and Modernization Act provision, which prohibits the US government from negotiating prices directly with drug companies^e^. The strength of the pharmaceutical lobby in the country is one of the explanations why Americans pay some of the world’s highest prices for their prescription drugs. The price escalation of patented medicines has a strong impact on health-care costs and restricts population access to more effective therapies. Because of ever-rising costs of biotechnology-derived drugs, Obama’s administration has sought to negotiate Medicare high-cost drug prices^f^. Nonetheless, the Pharmaceutical Research and Manufacturers of America (PhRMA), main lobbying group of the pharmaceutical industry, promptly and strongly reacted against this initiative to contain the soaring cost of health-care, and it is uncertain whether the US government will eventually succeed in the Congress.

**Figure:**
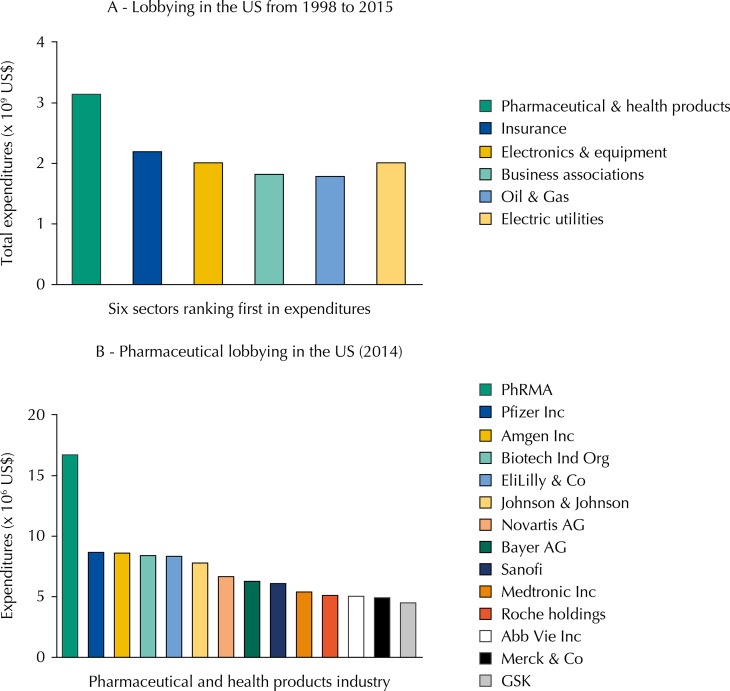
Expenditures of pharmaceutical and health product industry on lobbying activities in the US. As shown in panel A (upper part), pharmaceutical and health products industry ranked first among the six industry sectors that spent more money in lobbying activities between 1998 and 2015 (as to April 20th). Panel B (lower part) shows expenditures of some American drug and biomedicine organizations and companies in lobbying in 2014. The total expenditure of the pharmaceutical industry on lobbying amounted to US$230,932,063.00, and the number of registered lobbyists was 1,419. The Pharmaceutical Research and Manufacturers Association (PhRMA), main lobbyist organization in the US, occupies the top position. Data are from the US Senate Office of Public Records, retrieved from Center for Responsive Politics website on April 20, 2015 (www.opensecrets.org.lobby/top.php).

In Brazil, where expenditure on lobbying lacks transparency, the Pharmaceutical Research Industry Association (Interfarma)^f^, Brazilian equivalent of PhRMA, plays a major role in the advocacy of interests of major drug companies. Propaganda material published on its website indicates that Interfarma pursues a variety of private sector goals, such as^g^:

- Lowering taxes on medicines.
- Shortening the time taken by the regulatory agency (ANVISA) to approve marketing authorization for a new drug.
- Removing ‘administrative hurdles’ that prevent a prompt incorporation of new technologies into the Brazilian Unified Healthcare System (SUS).
- Combating counterfeit medicines, smuggling, and fiscal fraud.
- Protecting pharmaceutical products from theft and robbery.
- Lessening ‘bureaucratic constraints’ and redundancies in the ethical clearance system (CEP/CONEP/ANVISA) to speed up study protocol analysis and make the research approval process more flexible.

Interfarma industrious lobby has pressed ANVISA to shorten drug approval time and CONITEC (National Committee of Technology Incorporation into SUS) to be more flexible about cost-effectiveness criteria and to accelerate the incorporation of new drugs and medical technologies into the public health-care system. Interfarma and its affiliated drug companies are also close to achieving one of their greatest victories in Congress. In February 20, 2015, ANVISA published edits that state that, if it does not evaluate applications to conduct phase III trials within 90 days, and the ethical clearance system has approved the study protocol, automatically, applicants are to be considered as being authorized by the agency to initiate the clinical research^h^ ^,^ ^i^. Furthermore, a bill (PLS 200, 2015)^j^ that introduces profound alterations in the Brazilian ethical clearance system is now under consideration by the Congress. Although meeting most claims of major drug companies and of the Brazilian Association of Clinical Investigation, PLS 200/2015 was fiercely criticized by the National Committee of Research Ethics (CONEP), the Brazilian Association of Bioethics, and the Brazilian Association of Collective Health (ABRASCO)^k^. The criticisms addressed - but are not limited to – bill provisions that

- open up the possibility for using placebo-controlled groups even when there is an effective treatment for the medical condition, if a placebo group is necessary “to comply with a justifiable methodological requirement” (Art 27)^i^;
- exempt sponsors from the obligation to ensure post-trial treatment with no charge for patients if there is no risk of death or “clinically relevant worsening” of the disease, and if there is a “satisfactory therapeutic alternative” in the Country for the medical condition (Art 28)^i^;
- allow sponsors to offer money as an incentive for healthy volunteers to take part in phase I trials and drug bio-availability and/or bio-equivalence studies (Art 19 §2)^i^;
- create – in addition to institution ethical committees (CEP) – a new type of ethical committee (CI, independent ethical committee) that is not linked to a public or private institution;
- state that a single ethical review clearance by either a CEP or a CI is sufficient to begin a clinical study.

Another worrisome aspect is that the bill clause that addresses the protection of children and other vulnerable subjects (Art 21)^i^ is vague, thereby opening a door to interpretations of ethical principles that are less stringent than those that prevail in the US and EU. The bill requires an informed consent signed by children’s (or adolescents’) parents or legal representatives and states that “...study subjects’ desire to take part in the research or to withdraw from it should be respected, whenever he/she is capable to evaluate and decide with basis on the received information” (Art 21)^i^. In EU prevails the understanding that parents’ or legal representatives’ consent is insufficient and that adolescents above a certain age (13 to 15 years) must themselves be informed and formally consent, while younger children must assent to participate in the study^5^ ^,^ ^9^. For children under eight years of age, a specialized professional should assist investigators to interpret the children’s will to participate in the study. Moreover, unlike some trials with adults, ethical rules require that children or adolescents who are recruited to participate are suffering from the disease^5^ ^,^ ^9^. Participation of minors who will not potentially benefit from the intervention (i.e., recruitment of individuals based on altruism) is at best an ethically delicate topic and, in any case, must not involve a foreseeable risk or significant fear, pain, or discomfort^l^. The bill explicitly allows altruistic-based enrollment of children if the “clinical trial is essential for the population that the research individual represents” (Art 21)^I^, and makes no further remark regarding risks, discomfort, and participants’ will.

In the last years, Interfarma has lobbied for what it calls a “National Policy for Rare Diseases” (NPRD) in Brazil^m^. According to the Brazilian “National Policy of Integral Assistance to People with Rare Diseases”, rare diseases are those affecting 65 in 100,000 people^n^. Notwithstanding the unequivocal public health relevance of the theme, the pharmaceutical industry has its own interests in expanding the market for their “orphan drugs” in the country. Orphan drugs are medicines intended to treat rare diseases (for FDA, illnesses affecting < 200,000 people in the US) that sponsors are reluctant to develop under the usual marketing conditions. The orphan drugs status designated by US or EU agencies qualifies the sponsor of the product for government incentives that make their development more attractive. Pharmaceutical companies, however, have realized that, despite the small patient pool, the revenue potential of orphan drugs may offer great profitability if they are marketed at “the right price” across their lifetime^o^. In other words, these drugs can be highly profitable for pharmaceutical companies that adopt them. Interfarma proposals for a NPRD include “ensuring patient access to care and treatments”, “a differentiated registration mechanisms to accelerate the entry and sale of orphan drugs in the Brazilian market”, and “facilitating incorporation of orphan drugs into SUS”^k^. Interfarma identifies cost-effectiveness criteria currently employed as “barriers” to be overcome and suggests that the incorporation of orphan drugs into clinical protocols must be based on “clinical need” (i.e., “drugs must be considered clinically effective and necessary for treatment, but without meeting the criteria of cost-effectiveness”)^k^. Although maintaining that its proposal is viable, Interfarma anticipated that costs with orphan drugs would raise and affect public accounts^k^. The ongoing lobbying for NPRD illustrates that the pharmaceutical industry seeks to influence the establishment of health policy priorities and to align them with its own interests.

Like the wolf in sheep’s clothing of Aesop’s fable, pharmaceutical lobbying often hides its real intent under the guise of patients’ or doctors’ claims. It has become a common fact that patients’ organizations are in the forefront of lobbying campaigns for new drug approval and incorporation into SUS. Nonetheless, patients’ organizations generally rely on financial support from pharmaceutical companies, and such relationships are not fully transparent^6^ ^,^ ^7^. The extent to which the supporting industry influences the agenda and priorities of patient organizations is unclear^6^. At any rate, conflicts of interest and the various forms of collaboration between these unequal partners (one affluent and the other generally poor) raise justifiable concerns. Needless to say, medical associations’ positions about new medicines efficacy, safety, and cost-effectiveness can also be misdirected by drug industry lobbying^2^ ^,^ ^4^ ^,^ ^10^.

Furthermore, lobbying strategies often disguise drug companies’ real purposes by hiding them under the cover of a general claim of Brazilian society. Examples along this line are the Interfarma*-*sponsored “parliamentary study missions on innovation policies” to the US and the UK in 2011 (18 Congress members), 2012 (11 members), 2013 (7 members), and 2015^p^. The missions the alleged purpose of which was promotion of innovation in Brazil certainly paved the way for introducing PLS 200-2015 provisions that weaken the Country’s ethical requirements for clinical trials.

In conclusion, lobbying as such is not necessarily bad and at times it may be justifiable and needed (e.g., to defend minorities’ interests and rights against majority ruling). For democracy’s sake, however, it must be transparent, and a mandatory lobby register must be setup in the Country. Moreover, impacts of pharmaceutical lobbying on health policies, health-care costs, and clinical research regulation are topics of great importance for the Brazilian Unified Health System and should be included in the public health research agenda.

## Footnotes

a While lobbying invariably involves advocacy, advocacy does not necessarily involves lobbying. Nonetheless, the line between advocacy (not involving lobbying) and lobbying is so fine that at times it may be hard to distinguish them.

b Organisation for Economic Co-operation and Development – OECD. Lobbyists, government and public trust: promoting integrity by self-regulation. In: 405h Session of the Public Governance Committee; 23-20 Oct 2009; Paris. 2009 [cited 2015 Jul 26] (GOV/PGC (2009)9). Available from: http://www.oecd.org/officialdocuments/publicdisplaydocumentpdf/?doclanguage=en&cote=gov/pgc(2009)9

c Pinheiro W. Projeto de Lei do Senado Nº 336 de 2015. Disciplina a atividade de lobby ou de representação de interesses no âmbito dos órgãos e entidades dos Poderes da União, e dos órgãos e entidades da Administração Pública Federal, e dá outras providências. Brasília (DF): Senado Federal; 2015 [cited 2015 Aug 8]. Available from: http://www25.senado.leg.br/web/atividade/materias/-/materia/121578

d Lobbying Database [Internet]. Washington (DC): Center for Responsive Politics; 1980 - [cited 2015 Apr 20]. Available from: https://www.opensecrets.org/lobby/top.php?indexType=s

e Morgan D. Obama administration seeks to negotiate Medicare drug prices. Reuters edition US. Feb 2, 2015 [cited year Month day]. Available from: http://www.reuters.com/article/us-usa-budget-medicare-idUSKBN0L61OW20150202

f Interfarma, founded in 1990, is a non-profit organization with 54 affiliates among the biggest pharmaceutical companies that sell innovation drugs and generics in Brazil (http://www.interfarma.org.br).

g Interfarma. Cinco sugestões para melhorar já a saúde do Brasileiro. São Paulo: Interfama; [s.d.] [cited 2013 Sep 13]. Available from: http://www.sbmf.org.br/_pdf/biblioteca/14/5sugestoes_para_melhorar_ja_a_saude.pdf

h Ministério da Saúde, Agência Nacional de Vigilância Sanitária. RDC Nº 9, de 20 de fevereiro de 2015. Dispõe sobre regulamento para realização de ensaios clínicos com medicamentos no Brasil. *Diario Oficial Uniao*. 2015 Mar 3.

i Ministério da Saúde, Agência Nacional de Vigilância Sanitária. RDC Nº 10, de 20 de fevereiro de 2015. Dispõe sobre regulamento para realização de ensaio clínico com dispositivos médicos no Brasil. *Diario Oficial Uniao*. 2015 Mar 3.

j Amélia A, Moka W, Pinheiro W et al. Projeto de lei do Senado N 200, de 2015. Dispõe sobre a pesquisa clínica. Brasília (DF): Senado Federal; 2015 [cited 2015 Jul 25]. Available from: http://www25.senado.leg.br/web/atividade/materias/-/materia/120560

k Associação Brasileira de Saúde Coletiva. Projeto de lei do Senado nº 200, de 2015, retira da sociedade brasileira o controle das pesquisas envolvendo seres humanos. Rio de Janeiro: Abrasco; 2015 [cited 2015 Jul 24]. Available from: https://www.abrasco.org.br/site/2015/04/projeto-de-lei-do-senado-no-200-de-2015-retira-da-sociedade-brasileira-o-controle-das-pesquisas-envolvendo-seres-humanos/

l European Commission. Ethical considerations for clinical trials on medicinal products with the paediatric population: recommndations of the ad hoc group for the developing of implementing guidelines for Directive 2001/20/EC relating to good clinical practice in the conduct of clinical trials on medicinal products for human use: Final 2008 [cited 2015 Aug 6]. Available from: ftp://ftp.cordis.europa.eu/pub/fp7/docs/ethical-considerations-paediatrics_en.pdf

m Interfarma. Rare diseases: contributions for a national policy. São Paulo: Interfarma; [s.d.] [cited 2015 Jul 28]. (Special health editions, vol 5). Available from: http://www.interfarma.org.br/uploads/biblioteca/15-Rare%20Diseases%20-%20Site.pdf

n Ministério da Saúde. Portaria nº 199, de 30 de janeiro de 2015. Institui a Política Nacional de Atenção Integral às Pessoas com Doenças Raras. *Diario Oficial Uniao*. 2014 Feb 12;Section 1:44.

o EvaluatePharma. Orphan drug report 2014. London: Evaluate; 2014 [cited 2015 Aug 9]. Available from: http://info.evaluategroup.com/rs/evaluatepharmaltd/images/2014OD.pdf

p Wilson Center. The Brazilian Congress at the frontier of innovation. Washington (DC); 2014 [cited 2015 Jul 29]. Available from: https://www.wilsoncenter.org/publication/the-brazilian-congress-the-frontier-innovation
